# Too much sitting and all-cause mortality: is there a causal link?

**DOI:** 10.1186/s12889-016-3307-3

**Published:** 2016-07-26

**Authors:** Stuart J. H. Biddle, Jason A. Bennie, Adrian E. Bauman, Josephine Y. Chau, David Dunstan, Neville Owen, Emmanuel Stamatakis, Jannique G. Z. van Uffelen

**Affiliations:** 1 Active Living & Public Health, Institute of Sport, Exercise & Active Living (ISEAL), Victoria University, Footscray Park, Melbourne, VIC 8001 Australia; 2 Baker IDI Heart and Diabetes Institute, Melbourne, Australia; 3 University of Sydney, Sydney, Australia; 4 University of Queensland, Brisbane, Australia; 5 Monash University, Melbourne, Australia; 6 University of Melbourne, Melbourne, Australia; 7 Deakin University, Melbourne, Australia; 8 University of Western Australia, Perth, Australia; 9 The Australian Catholic University, Sydney, Australia; 10 Swinburne University of Technology, Melbourne, Australia; 11 University College London, London, UK

## Abstract

**Background:**

Sedentary behaviours (time spent sitting, with low energy expenditure) are associated with deleterious health outcomes, including all-cause mortality. Whether this association can be considered causal has yet to be established.

Using systematic reviews and primary studies from those reviews, we drew upon Bradford Hill’s criteria to consider the likelihood that sedentary behaviour in epidemiological studies is likely to be causally related to all-cause (premature) mortality.

**Methods:**

Searches for systematic reviews on sedentary behaviours and all-cause mortality yielded 386 records which, when judged against eligibility criteria, left eight reviews (addressing 17 primary studies) for analysis. Exposure measures included self-reported total sitting time, TV viewing time, and screen time. Studies included comparisons of a low-sedentary reference group with several higher sedentary categories, or compared the highest versus lowest sedentary behaviour groups. We employed four Bradford Hill criteria: strength of association, consistency, temporality, and dose–response. Evidence supporting causality at the level of each systematic review and primary study was judged using a traffic light system depicting green for causal evidence, amber for mixed or inconclusive evidence, and red for no evidence for causality (either evidence of no effect or no evidence reported).

**Results:**

The eight systematic reviews showed evidence for consistency (7 green) and temporality (6 green), and some evidence for strength of association (4 green). There was no evidence for a dose–response relationship (5 red). Five reviews were rated green overall. Twelve (67 %) of the primary studies were rated green, with evidence for strength and temporality.

**Conclusions:**

There is reasonable evidence for a likely causal relationship between sedentary behaviour and all-cause mortality based on the epidemiological criteria of strength of association, consistency of effect, and temporality.

**Electronic supplementary material:**

The online version of this article (doi:10.1186/s12889-016-3307-3) contains supplementary material, which is available to authorized users.

## Background

Sedentary behaviour is a collective term for behaviours undertaken in a seated or reclining posture during waking hours, with low energy expenditure (1–1.5 times resting metabolic rate) [1]. In practical terms, it is frequently referred to as ‘sitting time’ and this has recently generated widespread interest among health researchers, workplace managers and employees, as well as attracting significant media attention. Two main reasons for this interest are that, first, evidence is accumulating for the deleterious health effects of high levels of sitting and, second, distinguishing too much sitting from too little health-enhancing physical activity, or exercise, is a relatively novel approach from the traditional focus on only moderate-to-vigorous physical activity (MVPA). Importantly, excessive time spent in sedentary behaviours can co-exist in a lifestyle that might also include sufficient levels of MVPA [2, 3]. Hence, this suggests that for optimal health benefits, adults should both be physically active and limit their time spent sitting [4]. For these reasons, sedentary behaviour has become a new area of public health research and has begun to appear in public health guidelines, such as in the UK and Australia (see http://www.health.gov.au/internet/main/publishing.nsf/content/health-pubhlth-strateg-phys-act-guidelines).

After an initial focus on self-reported total sitting and total TV viewing time, and more recently, computer use and ‘screen time’, contemporary researchers have used objective monitoring tools (e.g., accelerometers and inclinometers) to estimate total time spent sedentary. Numerous studies and reviews, including those on young people and adults, have shown associations between either single self-reported sedentary behaviours (e.g., TV viewing) or objectively assessed total sedentary time and a number of health outcomes, including weight status, cardio-metabolic outcomes, mental health, and premature mortality (e.g., [5–12]).

In this context, there is the need to know whether current epidemiological evidence shows an association with health outcomes that is potentially causal. A frequently reported outcome in large-scale epidemiological studies is all-cause mortality (ACM), and in particular,, premature mortality. Other behavioural risk factors have been assessed for their association with ACM, including physical activity [13]. Moreover, a number of studies have addressed this for sedentary behaviour and several reviews have synthesised the evidence, as reviewed in this paper. However, as yet, none have systematically appraised evidence regarding whether sedentary behaviour may show the causal criteria proposed by Sir Austin Bradford Hill [14] (see [15, 16]). Although often stated as ‘criteria’, Bradford Hill actually never actually claimed that the nine factors he outlined were ‘criteria’, nor could they be “hard and fast rules of evidence that must be obeyed before we accept cause and effect” ([14], p.299) (see [17]). That said, criteria merely refer to something by which evidence is judged. In that sense, Bradford Hill did at least suggest considerations that could be used as ‘criteria’, although he said that “none of my nine viewpoints can bring indisputable evidence for or against the cause-and-effect hypothesis” ([14], p.299). Key factors for assessing causation from population observations are: strength of association, consistency, temporality, and dose–response. These, often alongside biological plausibility, are sometimes referred to as ‘Mill’s canons’ (see [18]), after John Stuart Mill’s writings on causal relationships in the 19^th^ century.

Additional considerations to the five listed above, Bradford Hill also proposed assessment of specificity, coherence, experimental evidence, and analogy. Further, in epidemiological studies, one may look by analogy at basic science or experimental studies, to examine whether there are putative biological mechanisms that might support the observed associations, and this is the Bradford Hill criterion of “biological plausibility”. Moreover, ‘coherence’ cannot be directly tested using epidemiological evidence. That said, coherence might be implicated, as could biological plausibility. Specificity may be judged as less relevant for the present analysis as there will be multiple causes of such an omnibus health marker as ACM [19]. Experimental evidence is not relevant to population-based observational epidemiological studies, while analogy is rarely considered (but is addressed in the Discussion section later).

Numerous appraisals of causality have been conducted in health research drawing upon Bradford Hill’s criteria (e.g., [20–22]), but none have addressed the health consequences of sedentary behaviours [see 10]. Dishman et al. [18], for example, analysed evidence on physical activity and ACM and concluded that there was strong evidence for a likely causal association.

Drawing upon Bradford Hill’s criteria, judgements in the current paper are made about:

- Strength of association: how strong or large is the association of sedentary behaviour with ACM? Is there a clinically meaningful difference in ACM between those exposed to higher levels of sedentary behaviour and those not?
- Consistency: how consistent is the association across different populations in different settings?
- Temporality: for mortality outcomes the exposure to high levels of sedentary behaviour must occur in advance of death. Longer periods of follow-up can strengthen the conclusion regarding causality as they reduce the risk of effects from co-morbidities. In addition, if primary studies include a sensitivity analysis, excluding the people who died in the first few years also strengthens the case for causality.
- Dose–response: do exposures to increasingly higher levels of sedentary behaviour lead to increasingly higher rates of ACM? Threshold or non-linear effects may also be present.

A great deal has been written about such judgements. While several authors have stressed the importance of Bradford Hill’s considerations, there is also the view that they are not absolute, should be “viewed as aids to judgement, not as arbiters of reality” ([23], p.794), and will inevitably leave room for choices and preferences in how the traditional factors are selected and used [24]. Therefore, in this study we adopted the novel approach of examining evidence on the relationship between sedentary behaviour and ACM, through the use of a traffic light system (see Methods). Specifically, we assessed published systematic reviews and appraised all primary papers from those systematic reviews that were suitable for analysis.

## Methods

### Literature search

PubMed was searched up to March 2015 to identify systematic reviews and meta-analyses examining relationships of sedentary behaviours with ACM. Groups of thesaurus terms and free terms for mortality, sedentary behaviour (e.g., sitting, sedentary, television, occupational activity) and publication type (e.g., review, meta-analysis) were used. This resulted in the following search: (mortality [MeSH] OR mortality [tiab]) AND (sitting [tiab] OR sedentary [tiab] OR tv [tiab] OR television [tiab] OR (occupational activity [tiab])) AND (review [pt] OR review [tiab] OR meta-analysis [pt] OR meta-analysis [tiab]). Additional reviews and meta-analyses were identified by manually checking the reference lists of included papers and searching the authors’ own literature databases.

To be included in the present analysis, review papers had to meet the following criteria: 1) population to include adults (usually those at least 18 years of age, sometimes including those 16 years and over); 2) include at least one measure of sedentary behaviour; 3) report associations of sedentary behaviours with all-cause mortality; and, 4) be a systematic review or a meta-analysis. Reviews summarizing or quantifying the evidence for associations could be based on subjective (e.g., questionnaires) or objective measures of sedentary behaviour (e.g., accelerometers, inclinometers), as well as overall sedentary behaviour or setting-specific sedentary behaviour (e.g., occupational sitting, TV viewing time). Reviews or meta-analyses including measures of sedentary behaviour that were a combination measure of sedentary behaviour and physical activity, such as categorical measures with sedentary as the least active category, were excluded.

**Fig. 1:**
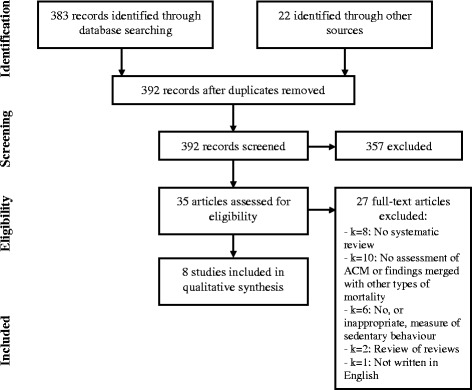
PRISMA flow chart showing identification, screening, and selection of systematic review papers

Only full text peer reviewed articles written in English were considered for inclusion. Titles and abstracts of the identified references were reviewed to exclude articles out of scope. Subsequently, two reviewers independently reviewed the full text of all potentially relevant references for eligibility. Disagreements between these reviewers were discussed with a third reviewer and a consensus decision was reached. Primary studies included in all of the reviews were also scrutinized to allow for additional appraisal of causality. Of 19 primary studies, two [25, 26] were excluded for assessing sedentary behavior inappropriately (i.e., as low physical activity), and one study [27] was assessed separately for two sedentary measures (i.e., total sitting and TV time). Overall, therefore, this left 18 primary studies for review (see the PRISMA flowchart in Fig. 1 for the review papers, and also Additional file 1: Table S1 & Additional file 2: Table S2) [27–43].

### Criteria for rating systematic reviews and individual studies

The systematic reviews and primary studies were assessed for evidence concerning strength of association, consistency, temporality, and dose–response. To provide an interpretable and practical assessment, we adopted a traffic light system (i.e., green, amber and red). Green indicated that there was evidence for causality; amber indicated inconclusive evidence for causality; red indicated no evidence for causality (either evidence is null or there is lack of evidence).

#### Ratings for systematic reviews

The factors used to assess causality for each systematic review were:

1. Strength of association: not significant = red; small (5-10 % increased risk), but significant = amber; large and significant (>10 % increased risk) = green.
2. Consistency: if individual studies represented a range of countries, age groups and sex = green; if the subgroup analysis showed similar results = green. More limited sampling was rated amber.
3. Temporality: proportion of included studies with follow up > 6 years. If more than 50 % = green, otherwise amber. If all are < 2 years follow up = red.
4. Dose–response: used categorical data. 2 categories only = red; 3 categories and significant difference between the categories = green; > 3 categories and significant differences across the majority of the categories = green.

#### Ratings for primary studies

The factors used to assess causality for each primary study were:

1. Strength of association: reported results of fully adjusted model and Hazard Ratios or Odds Ratios with 95 % confidence intervals (categorical data, not significant = red, 5-10 % increased relative risk in “high” sedentary behaviour group = amber; >10 % increased relative risk = green).
2. Consistency: if nationally representative sample = green; if subsample, certain age group, or one sex = amber. Absolute judgements on representativeness are not possible, but ‘nationally representative’ required coverage across a country and not be restricted regionally.
3. Temporality: if maximum potential follow-up <2 years = red; 3-6 years amber; > 6 years = green.
4. Dose–response: study had to use categorical data. For 2 categories only = red; 3 categories and significant difference between both categories and the reference group = green; > 3 categories and significant differences between the majority of the categories = green.

An overall rating for likely causality was derived for each systematic review and primary study. This was done by assigning a colour code based on the majority coding across the four Bradford Hill factors. In the event of the review or study have two ratings for each of two factors (always green and amber), the final rating was based on the colour coding for strength of association. Each systematic review and primary study was assessed by two reviewers independently with reference to the assessment criteria. Assessments were presented at a meeting where a third assessor was present to discuss and assist in resolving any discrepancies.

## Results

Potentially relevant articles (*n* = 392) were identified in the search. After excluding the records out of scope, we checked 35 articles in full. Of these, 27 did not meet the inclusion criteria (see Fig. 1). After excluding these 27 papers, eight reviews meeting the inclusion criteria were included in the analysis [7, 8, 44–49]. These varied in size from focused reviews only of TV viewing with three primary studies [45] to the assessment of total sedentary time with 12 papers [49].

Sedentary behaviours were operationally defined in various ways, including TV viewing time, screen time, and total sedentary (sitting) time. One review focused only on older adults [48] and one review synthesised only studies that adjusted for physical activity [49]. Most primary studies appeared in more than one review. Eleven of these primary papers appeared in less than four reviews, while one paper [28] was included in six reviews. No primary papers appeared in all eight reviews (see Additional file 1: Table S1).

With reference to Bradford Hill’s criteria, each of the eight systematic reviews was assessed for evidence on strength, consistency, temporality, and dose–response (see Additional file 1: Table S2). Each potential causal criterion within each review was judged and given a colour code, as described. Each review was then colour coded for its overall assessment of causality. On this basis, five of the eight reviews were rated green, with three rated amber. For strength of association, four (50 %) were rated green and four amber, for consistency seven (88 %) were green, for temporality six (75 %) were green, and for dose–response five (63 %) were red. These assessments suggest that at the level of systematic review data, there is support for the conclusion that epidemiological studies of sedentary behaviour show a likely causal association with all-cause mortality. This is particularly supported through evidence on consistency and temporality, with less conclusive evidence for strength of association. There was no consistent evidence for a dose–response effect and less research overall that addressed dose–response associations between sedentary behaviour and ACM.

Additional file 3: Table S3 shows the analysis for each of the causality factors at the level of primary studies. Overall, 12 (67 %) primary studies were rated green, 6 (33 %) amber and none red. Of the 18 studies, 13 (72 %) showed evidence for strength of association (green) and 12 (67 %) for temporality. Ratings for consistency (k = 13, 72 % amber) and dose–response (k = 10, 56 % amber and k = 2 red) were less supportive of a causal association. These assessments suggest that at the level of primary studies included in the systematic reviews, there is reasonable support for the conclusion that sedentary behaviour is causally associated with all-cause mortality. This is supported primarily through evidence concerning strength of association and temporality, with less conclusive evidence for consistency or a dose–response effect.

## Discussion

There is reasonable epidemiological evidence for a causal relationship between sedentary behaviour and all-cause mortality. Within the colour-coding classification used, the eight systematic reviews examined showed clear support for two of the four Bradford Hill factors for assessing causation from population observations: consistency (7 green) and temporality (6 green). There was some support, though less consistent, for strength of association (4 green) and no support for dose–response (5 red). Only three reviews provided data on dose–response relationships. When assessing reviews as a whole, five of the eight reviews were rated green, indicating support for a causal relationship between sedentary behaviour and ACM. Of the 17 primary studies (18 assessments), 67 % were rated green, with evidence for strength and temporality.

### Assessing causality using the Bradford Hill framework

Within epidemiological research, judging strength of association is not easy. Complicating our assessments was that the reviews and primary studies we examined differed by how they analysed data, and different measures of sedentary behaviour were often adopted. Rosenthal [50] has suggested that an odds ratio between 1.5 and 2.5 could be considered of ‘moderate’ strength, but no criteria are provided for hazard ratios. Of the six reviews providing summary data on strength of association, none reached the level of ‘moderate’ strength when comparing highest versus lowest sedentary categories. However, three of the six were close to such a value, showing relative risk ratios between 1.45-1.49. These values are slightly less than what Khaw et al. [51] reported for mortality for smoking (*RR* = 1.77), comparable to five or more daily servings of fruit and vegetables (RR = 1.44), but higher than being physically inactive (RR = 1.24) and consuming more than 14 units of alcohol per week (RR = 1.26). These comparisons are from analyses of 20,244 men and women in England aged 45–79 years in 1993–1997 and followed up to 2006.

In a recent analysis of the Australian ‘45 and Up’ study, Ding and colleagues [52] calculated hazard ratios for ACM for seven risk factors singly and in combination. The HR for prolonged sitting, when analysed singly, was 1.15. Interestingly, again when considered singly, this was the most prevalent risk factor. But this HR value is small and was less than for physical inactivity alone (HR = 1.61) but higher than poor diet (HR = 1.04).

It is debatable how strong an association we might expect for the behaviour of ‘sitting’ on ACM, but the association that often appears for sitting is sometimes comparable to other risk factors. For example, Bouchard et al. [4] plot the relative risk for ACM for sitting (from one large study), cardiorespiratory fitness, and MVPA. The trajectories are similar although they reported that while all three have strong associations with premature mortality, the strongest effects are from fitness. It is also possible to argue that sedentary behaviour, even with a slightly smaller effect on ACM that fitness or MVPA, will affect a large proportion of the population, thus making it an important public health issue. This is due to the high volume of total daily sitting for large numbers of people.

Judgments of consistency show that much of the evidence concerns white European and ‘western’ populations, with just one large study from Asia, and none from Africa or South American. There seem to be no obvious differences by sex at the level of reviews, but two primary studies showed contradictory findings with one reporting stronger effects for women [32] and one for men [29]. This may be a function of ethnicity with the studies reporting data for US and Japanese adults. That said, a pooled analysis of multiple cohorts from England and Scotland with over 11,000 adults, showed ACM to be associated with occupational sitting for women only [53]. To strengthen judgements concerning the consistency of evidence, studies are required with more diverse populations using standardised assessments of sedentary behaviour.

The assessment of temporality showed support for the notion of a causal link between sedentary behaviour and ACM. The majority of primary studies had follow-up periods of greater than 7 years, and only three studies had less than 3 years, although the evidence from shorter follow-up studies may not be ready for publication. Most studies we reviewed reported that they did not analyse data for those dying within 2 years of sedentary behaviour assessment thus avoiding the potential confounding influence of occult disease.

Most reviews and primary studies did not report data addressing dose–response effects. From the three studies where data were available, it appears that any effects on ACM are from much higher levels of sedentary behaviour. For example, the review concerning TV viewing by Grontved and Hu [45] showed that only from around 3 h per day of TV was there an increase in mortality. Similarly, Katzmarzyk and Lee [46] showed the greatest effects for ACM for the third tertile for both total sitting and TV viewing. Such findings suggest a non-linear or even a threshold effect for sedentary behaviour on ACM, with lower levels of the behaviour having little effect. Indeed, Chau et al’s [47] analysis of six studies concluded that the risk of ACM increased significantly from about 7 h per day of sitting. Further work on dose–response relationships is required, including analyses of total sedentary time and specific behaviours, such leisure-time TV viewing, workplace sitting and car travel. For now, we cannot conclude with great precision about dose–response effects as most studies (primary and reviews) did not address this (but see [4]).

Biological plausibility could not be directly assessed in our analyses as we were reviewing only epidemiological studies. However, it can be argued at a general level that higher rates of sitting are plausibly associated with poor health, and this can include an analysis of the factor of ‘analogy’ proposed within the Bradford Hill framework. It has been argued that analogies may contribute to assessments of the weight of evidence [23]. Moreover, there are at least three ways in which analogies could be drawn when investigating sedentary behaviour and health outcomes. First, there is convincing evidence that low levels of physical activity are associated with ACM [13]. This has been shown for men and women when comparing multivariate adjusted differences in ACM between those reporting no physical activity and those doing ‘light’ physical activity. While it has been argued elsewhere that ‘sitting’ is not the same as avoiding physical activity (e.g., someone could stand all day but still not move too much) [2, 5], the argument by analogy is persuasive. Indeed, reductions in sitting time are most likely to transfer into greater amounts of light physical activity, and the meta-analysis by Löllgen et al. [13] shows that this could be important for ACM. Second, there is evidence that time spent standing is associated in a dose–response manner with ACM [54]. Standing, and other forms of light movement, are strongly negatively associated with sedentary behaviour [55].

Third, there is a long history in physiology showing rapid and detrimental effects on health and functional markers from extended bed rest [56] and from hind limb unloading in rodents [57, 58]. Finally, evidence on the effects of weightlessness in space, and the associated deleterious health effects, has been suggested as a clear analogy to the low musculo-skeletal loading of prolonged sitting [59]. Collectively, these types of studies have provided unique insights into the potential causal relationship and possible underlying mechanisms through which physical inactivity may contribute to chronic disease development. Recent reviews have documented evidence on the many physiological responses related to imposed physical inactivity [56, 60], including a reduced capacity to use fat as a substrate or ATP production, muscle atrophy, a shift in muscle fibres toward fast-twitch glycolytic type, muscle insulin resistance, ectopic fat storage and increased central and peripheral adiposity. In summary, while not all of these fields address ACM, there is a case for ‘analogy’ and ‘biological plausibility’ concerning significant negative health effects of sedentary behaviour from related fields.

While the initial evidence on potential biological mechanisms of poor health markers from sedentary behaviour is largely derived from animal studies [61], emerging human research is promising, notwithstanding that such studies do not assess mortality outcomes [62, 63].

### Does physical activity matter?

It is important for future studies to control for potential confounding variables in any assessment of links between sedentary behaviour and health outcomes. One such key variable is MVPA. While it may not be too surprising to see the positive effects of physical activity over-power any effects for sitting time, it should be noted that when assessed by accelerometry, large proportions of the adult population have low levels of physical activity [64], thus sedentary behaviour is still an important behaviour to address for the majority and thus is a significant public health issue.

Nevertheless, there is still some debate if, and by how much, sedentary behaviour is associated with deleterious health outcomes independently of MVPA. For example, while many studies show associations between sedentary behaviour and poor health following adjustment for MVPA levels – indeed many studies did this in their fully adjusted models – the most recent and comprehensive of the systematic reviews [49] showed that the link between sedentary behaviour and ACM held only for those with low levels of physical activity. In addition, Bjork-Petersen et al. [65] have reported on a large study of Danish adults and found that being physically inactive during leisure time but also sitting more than 10 h per day was associated with increased risk of ACM, as one might expect. However, for those who were physically active, sitting time had minimal effect on ACM. This is similar to an analysis of cardiovascular disease outcomes in a large study of sitting time and physical activity in women [66]. Specifically, more time spent sitting increased the risk of CVD except for those classified as the most active.

A recent analysis of the Whitehall II cohort has suggested that sedentary behaviour and ACM are not associated [67]. However, the sample was a large group of London ‘civil servants’ who were free from CVD and likely to be considered quite healthy. They were not obese and had high levels of physical activity. Moreover, the average age at baseline was in the mid-40s, which may be too young to show any significant mortality effects. In summary, this null finding may be the result of the population studied, but also includes the possible explanation that their physical activity levels partially attenuated the negative health effects of prolonged sitting.

In a novel analysis of over 200,000 mid- and older-aged adults in Australia [68], analyses investigating the ‘effects’ of substituting certain behaviours for others showed “an independent beneficial effect of standing on mortality (3 % decrease in risk per hour of standing in the whole sample), and this association was present in both those who met and did not meet the physical activity recommendations. These beneficial associations of standing with mortality were even more substantial (5 % decrease in risk for each hour of sitting replaced with standing) when standing time displaced sedentary behaviour” (p. 8). These findings suggest that reductions in sedentary behaviour are important for improved mortality outcomes. Additionally, recent evidence by Healy et al. [63], also using isotemporal substitution models but with one cross-sectional population data set, showed that replacing sitting with standing was likely to have significant cardiometabolic-related health benefits. Replacing sitting with stepping was more beneficial for adiposity outcomes.

Clearly, more needs to be known about the interaction of sedentary behaviour and physical activity. This may require an analysis of the role of MVPA as a moderator rather than just adjusting for levels of MVPA and taking into account co-exiting behaviours, including sleep [69, 70]. Accounting for these behaviours across 24 h may require investigation using compositional data analysis [70, 71]. This is clearly an important future direction.

### Measurement of sedentary behaviour

The studies that we examined have several measures of sedentary behaviour exposure – the majority of which were derived from self-report assessment instruments. Television viewing time has consistently emerged as a strong exposure variable, although it is only a subset of overall sedentary behaviour. One of the reasons why this measure has performed so strongly in epidemiological studies is likely to be that recall of television viewing time – typically a consistently repeated habitual behaviour – provides an exposure measure that may be less strongly influenced by measurement error than the other indices that have been used. However, TV time may be seriously confounded by socio-economic status and by unhealthy dietary snacking behaviours concomitant with television time. In addition, self-reported overall sitting time is a consistent predictor of mortality in prospective epidemiological studies, as shown. While the precision of people’s recall of overall daily or weekly sedentary time is likely to be limited, these measures are valuable in large population studies in that they provide a rank of individuals on their total exposure to sedentary behaviour [72].

The measurement of sedentary behaviour is a moving target – the rapid uptake of cheap, convenient and easy-to use wearable and screen-based devices provides a plethora of new opportunities for sedentary time. Older studies focused primarily on TV viewing time while new studies address ‘screen time’. Ongoing epidemiological studies may soon provide a large body of findings with exposure measures of sedentary behaviour that are derived from small, unobtrusive wearable devices (including accelerometers that capture movement and inclinometers that assess posture directly). Data from these devices will allow not only objectively-assessed overall sedentary time to be employed as an exposure variable more readily, they will also provide the capacity to examine several important and as yet unexplored questions on patterning of sedentary behaviours. Examples include how sitting time is accumulated in different bout lengths, the potential protective role of regularly breaking up sedentary time, and how bouts of physical activity may be protective.

The limitations of our analysis include the usual methodological limitations of observational epidemiological data, and thus excludes a direct analysis of experimental studies that would contribute to biological plausibility. The reliance on self-report exposure measures has potential measurement as well as non-specific and specific unmeasured confounding. Not all studies assessed sedentary behaviour or physical activity in the same way, and some did not assess diet at all. One issue is the extensive use of TV viewing as a proxy measure of sedentary behaviour, when total sitting time may show a direct socio-economic gradient, compared to the inverse gradient seen for TV time alone. [73]. In addition, our use of the colour coding system, while designed to be helpful to the reader, inevitably relies on subtle judgements and the criteria specified for assessment.

## Conclusions

While research on sedentary behaviour and health outcomes is still at an early stage, our analysis of the evidence of the relationship with all-cause mortality appears to be reasonably consistent when considered in the light of the well-known factors used to judge causal relationships using Bradford Hill’s framework. Prolonged periods spent sitting are ubiquitous in the workplace, through time spent sitting in cars, and through screen-based entertainment in home environments. Addressing too much sitting in the context of public health approaches to chronic disease prevention, occupational health and clinical practice will require innovative approaches that may be different to those used to promote exercising and physical activity. From the rapidly developing body of evidence that we have examined, there may emerge novel, feasible, and sustainable approaches to population health improvement. However, such new opportunities should not distract from a continuing emphasis on moderate-to-vigorous physical activity and the broader array of bodily movements and exercises that are crucial to the maintenance of metabolic and musculoskeletal health and mental well-being, particularly in ageing populations. Increases in physical activity and reductions in sedentary behaviour are clearly warranted for public health.

## Additional files

Additional file 1: Table S1. Characteristics of systematic reviews with primary papers included and causality colour coding for both systematic reviews and primary study papers^a^. (DOCX 25 kb) Additional file 2: Table S2. Analysis of causality across systematic reviews. (DOCX 28 kb) Additional file 3: Table S3. Analysis of causality across primary studies included in systematic reviews. (DOCX 31 kb)
