# Supplementary material for: Too much sitting and all-cause mortality: is there a causal link?
Source: BMC Public Health. 2016 Jul 26;16:635. doi: 10.1186/s12889-016-3307-3 (PMC4960753; doi:10.1186/s12889-016-3307-3)
Supplement: Additional file 1: Table S1. — Characteristics of systematic reviews with primary papers included and causality colour coding for both systematic reviews and primary study papersa. (DOCX 25 kb) [file 12889_2016_3307_MOESM1_ESM.docx]

Table 1. Characteristics of systematic reviews with primary papers included and causality colour coding for both systematic reviews and primary study papers^a^.

|  | **Systematic Reviews (k=8)** | | | | | | | |
| --- | --- | --- | --- | --- | --- | --- | --- | --- |
|  | **Proper et al. (2011)** | **Thorp et al. (2011)** | **Grontved & Hu (2011)** | **Katzmarzyk & Lee (2012)** | **Wilmot et al. (2012)** | **Chau et al. (2013)** | **Rezende et al. (2014)** | **Biswas et al. (2015)** |
| Number of primary studies in review (number included) | K=3 (2) | K=6 | K=3 | K=5 | K=8 | K=6 | K=4 | K=13 (12) |
| Sedentary behaviour addressed as stated in review | ‘prospective studies of sedentary behaviour’ | ‘longitudinal studies of sedentary behaviour ’ | ‘TV viewing’ | ‘sitting time’; ‘TV viewing’ | ‘sedentary behaviour’ | ‘total daily sitting time’ & ‘TV viewing’ | ‘sedentary behaviour in older adults’ | ‘sedentary time’ (and adjusted for PA) |
| Conclusion from review | “strong evidence was  found for sedentary behavior to be related to all-cause … mortality” | “sedentary behavior has  been shown to be consistently associated with increased  risk for all-cause … mortality” | “prospective studies  suggest that longer duration of TV  viewing time is consistently associated  with higher risk of … all-cause mortality” | “extended sitting time and television viewing may  have the potential to reduce life expectancy in the USA” | “greater  sedentary time is significantly associated with an increased  risk of all-cause mortality” | “higher amounts of daily total  sitting time are associated with greater risk of dying from all causes” | “This review confirms previous evidence of the relationship  between sedentary behavior and all-cause mortality among (older)  adults” | “sedentary time … was  independently associated with a greater risk for all-cause  mortality” |
| Dates of search | 1989-2010 | 1996-2011 | 1970-2011 | Full database search through to 2011 | 1980-2012 | 1989-2011 | Full database search through to 2013 | Full database search through to 2014 |
| Primary study papers included & causality colour coding*** |  |  |  |  |  |  |  |  |
| Weller & Corey 1998 |  |  |  |  | ✓ |  |  |  |
| Inoue et al 2008 |  | ✓ |  |  | ✓ | ✓ |  | ✓ |
| Katzmarzyk et al 2009 | ✓ | ✓ |  | ✓ | ✓ |  |  | ✓ |
| Dunstan et al 2010 | ✓ | ✓ | ✓ | ✓ | ✓ |  |  | ✓ |
| Patel et al 2010 |  | ✓ |  | ✓ | ✓ |  |  | ✓ |
| Wijndaele et al 2010 |  | ✓ | ✓ | ✓ | ✓ |  |  |  |
| Stamatakis et al 2011 |  | ✓ | ✓ | ✓ | ✓ |  |  | ✓ |
| Matthews et al 2012 |  |  |  |  | ✓ | ✓ |  | ✓ |
| Van der Ploeg et al 2012 |  |  |  |  |  | ✓ |  | ✓ |
| Koster et al 2012 |  |  |  |  |  | ✓ |  | ✓ |
| Pavey et al 2012  [full published citation now 2015] |  |  |  |  |  | ✓ | ✓ | ✓ |
| Campbell et al 2013 |  |  |  |  |  |  | ✓ |  |
| Chau et al 2013 |  |  |  |  |  | ✓ |  |  |
| Kim et al 2013 |  |  |  |  |  |  |  | ✓ |
| Leon-Munoz et al 2013 |  |  |  |  |  |  | ✓ | ✓ |
| Martinez-Gomez et al 2013 |  |  |  |  |  |  | ✓ |  |
| Seguin et al 2014 |  |  |  |  |  |  |  | ✓ |
| **Papers excluded from present analysis ^b^** |  |  |  |  |  |  |  |  |
| George et al 2013 |  |  |  |  |  |  |  | ✓ |
| Graff-Iversen et al 2007 | ✓ |  |  |  |  |  |  |  |
| Review summaries | Green=2 (100%) | Green=4 (67%)  Amber=2 (33%) | Green=2 (67%)  Amber=1 (33%) | Green=4 (80%)  Amber=1 (20%) | Green=5 (63%)  Amber=3 (37%) | Green=3 (50%)  Amber=3 (50%) | Green=3 (50%)  Amber=3 (50%) | Green=9 (75%)  Amber=3 (25%) |

Notes:

1. The traffic light coding for each review is based on the 4 factors for assessing causality, not the individual studies listed here (see Table 2).
2. Papers in original systematic reviews but excluded from present analysis as they were judged not to assess sedentary behaviour.

*** Number of papers included >6 reviews = 0; in 6 reviews = 1; in 5 reviews = 2; in 4 reviews = 3; in <4 reviews = 11.
