# Supplementary material for: Too much sitting and all-cause mortality: is there a causal link?
Source: BMC Public Health. 2016 Jul 26;16:635. doi: 10.1186/s12889-016-3307-3 (PMC4960753; doi:10.1186/s12889-016-3307-3)
Supplement: Additional file 2: Table S2. — Analysis of causality across systematic reviews. (DOCX 28 kb) [file 12889_2016_3307_MOESM2_ESM.docx]

Table 2. Analysis of causality across systematic reviews

| **Authors and date** | **Strength** | **Consistency** | **Temporality** | **Dose-response** |
| --- | --- | --- | --- | --- |
| Overall rating for review below: |  |  |  |  |
| Proper et al. (2011) | More SB associated with increased mortality: based on the fındings of the two high-quality studies,  there is strong evidence for a relationship between sedentary  behaviour and ACM | 2 studies from Western/European populations (Australia k=1; Canada k=1)  Age range: all adult ages included  Sex: Male and female (k=2)  Large samples from Australia and US | 1 out of 2 studies FU more than 7 years | Not examined |
| Overall rating for review |  |  |  |  |
| Thorp et al. (2011) | 5/6 studies showed an increased risk of ACM and time spent in SB. | Large population based samples  6 studies from predominantly white ‘Western/European’ populations (Australia k=1; US k=1) Japan k=1, Canada k=1, UK k=2).  Age range: all adult ages included.  Sex: all men/women | 4 out of 6 studies had follow-up > 7 years. | Not examined |
| Overall rating for review |  |  |  |  |
| Grontved & Hu (2011) | TV >2hrs/day (adjusted for PA in only 1 of 3) associated with increased mortality risk: pooled HR = 1.13 (95% CI 1.07-1.18) | 3 studies from predominantly white ‘Western/European’ populations (Australia k=1; UK k=2)  Age range: all adult ages included.  Sex: all male & female (k=3) | 1 out of 3 studies had follow-up > 7 years. | Significant non-linear relationship.  Graphed data show increasingly elevated risk from 3hrs TV/day |
| Overall rating for review |  |  |  |  |
| Katzmarzyk & Lee (2012) | More SB associated with increased mortality risk:  Total sitting pooled RRs:  Level 1 (ref): 1.0  Level 2: 1.18 (1.14-1.21)  Level 3: 1.45 (1.39-1.51)  TV viewing pooled RRs:  Level 1 (ref): 1.0  Level 2: 1.17 (1.04-1.32)  Level 3: 1.49 (1.22-1.82). | Total sitting: 2 studies from predominantly white Western/European populations (North America k=1, Canada, k=1)  Age range: all adult ages included  Sex: Male and female (k=2)  TV viewing: 3 studies from predominantly white Western/European populations (Australia k=1; UK k=2)  Age range: all adult ages included  Sex: Male and female (k=3) | Total sitting: 2 out of 2 studies FU more than 7 years  TV viewing: 1 out of 3 studies FU more than 7 years | Evidence for dose-response relationship:  Total sitting pooled RRs:  Level 1 (ref): 1.0  Level 2: 1.18 (95% CI 1.14 to 1.21)  Level 3: 1.45 (95% CI 1.39 to 1.51)  TV viewing pooled RRs:  Level 1 (ref): 1.0  Level 2: 1.17 (95% CI 1.04 to 1.32)  Level 3: 1.49 (95% CI 1.22 to  1.82). |
| Overall rating for review |  |  |  |  |
| Wilmot et al. (2012) | More SB associated with increased ACM risk:  Pooled RRs for all studies were 1.49 (95% CrI 1.14-2.03)    5 studies adjusted for PA : 1.40 (0.45-3.82) | Large population based samples  8 studies from predominantly white ‘Western/European’ populations (Australia k=1; US k=2) Japan k=1, Canada k=2, UK k=2).  Age range: all adult ages included.  Sex: Male & female =7, female only =1 | 6 out of 8 studies had follow-up > 7 years. | Not examined |
| Overall rating for review |  |  |  |  |
| Chau et al. (2013) | More SB (adjusted for PA) associated with increased mortality risk:  pooled HR = 1.02 (1.01-1.03) for each additional hour | 6 studies from predominantly white ‘Western/European’ populations (Australia k=2; Norway k=1; US k=2) plus 1 from Japan.  Age range: all adult ages included.  Sex: Male & female k=5; female-only k=1 | 2 out of 6 studies had follow-up > 7 years. | Not a linear trend.  Adjusted model: For every 1-hour increment  of sitting:  0-3h/day:  HR=1.00 (95% CI: 0.98-1.03);  >3-7h/day: HR=1.02 (95% CI: 0.99-1.05);  >7h/day total: HR=1.05 (95% CI: 1.02-1.08). |
| Overall rating for review |  |  |  |  |
| Rezende et al. (2014) | Based on the fındings of 4 studies, all showed increased risk of ACM in the highest sitting group. | Older adults >60  Western/European’ populations (Australia k=1; Spain k=2; US k=1)  Spain: not population based studies  Sex: all males & females (k=4) | All 4 studies  had follow-up > 7 years. | Not examined |
| Overall rating for review |  |  |  |  |
| Biswas et al. (2015) | More SB (adjusted for PA) associated with increased mortality risk:  pooled HR = 1.24 (1.09–1.41)  Association between SB and ACM was not significant for those with high PA levels: pooled HR, 1.16 (0.84-1.59).  Association between SB and ACM was stronger in those with low PA levels: pooled HR = 1.46 (1.22-1.75).  Sensitivity analysis did not change outcome. | 12 studies from predominantly white ‘Western/European’ populations (North America k=6; Australia k=3, Europe k=2) plus 1 from Japan.  Age range: all adult ages included.  Sex: Male & female k=9; female-only k=4 | 7 out of 12 studies had follow-up > 7 years. | Not examined |
| **Overall ratings by criteria**:  Green: 5  Amber: 3  Red: 0  Summary rating: Green | Green: 4  Amber: 4  Red: 0  Summary rating: Green | Green:7  Amber: 1  Red: 0  Summary rating: Green | Green: 6  Amber: 2  Red: 0  Summary rating: Green | Green: 2  Amber: 1  Red: 5  Summary rating: Red |

Notes:

1. Note: deleted primary studies are not included in assessments, except where composite assessments precluded this (e.g., strength and dose-response).

Abbreviations: ACM: all-cause mortality; CI: confidence interval; CrI: Bayesian equivalents of 95% CI; HR: hazards ratio; PA: physical activity; RR: relative risk ratio; SB: sedentary behaviour
