# Supplementary material for: Too much sitting and all-cause mortality: is there a causal link?
Source: BMC Public Health. 2016 Jul 26;16:635. doi: 10.1186/s12889-016-3307-3 (PMC4960753; doi:10.1186/s12889-016-3307-3)
Supplement: Additional file 3: Table S3. — Analysis of causality across primary studies included in systematic reviews. (DOCX 31 kb) [file 12889_2016_3307_MOESM3_ESM.docx]

Table 3. Analysis of causality across primary studies included in systematic reviews

| **Authors and date** | **Strength** | **Consistency** | **Temporality** | **Dose-response** |
| --- | --- | --- | --- | --- |
| Overall rating for study below: |  |  |  |  |
| Weller & Corey 1998 | Compared to > half of the time: OR 0.84 (95%CI 0.64-1.11) for half the time;  0.58 (0.44-0.75) for <half of the time.  Not described if analysis was repeated excluding people who died in first years. | Canadian women aged 60+ years (n=6,620)  Adjustment for marital status, education, income, self-rated health, smoking did not affect OR. | 7y FU. | Only 3 categories; and only lowest category significantly different from reference category. |
| Overall |  |  |  |  |
| Inoue et al. 2008 | Men: compared to <3 hrs, HR for 3-8 hrs 1.02 (0.95–1.11);  >= 8 hrs 1.18 (1.04–1.35).  p for trend =0.036  Women: compared to <3 hrs, HR for 3-8 hrs 0.95 (0.85–1.06);  >= 8 hrs 1.10 (0.82–1.25).  p for trend =0.698  Excluded deaths in first 3 yrs in PA analyses, not reported for sitting. | Japanese men and women aged 45-74y (n=83,034).  Effects stronger in men than women.  Adjusted for age, area,  occupation, history of diabetes, smoking, alcohol  intake, BMI, energy intake, heavy physical work or strenuous exercise, walking/ standing hours, leisure-time sports or physical exercise.  Analyses stratified by sex. | 8.7y FU. | Only 3 categories. Effect significant only for >8h/d. Dose-response shown for men only. |
| Overall |  |  |  |  |
| Katzmarzyk et al. 2009 | HR 1.54 for ‘almost all’ sitting vs. none. | Canadian men and women (n=17,013). SB adjusted for LTPA, age, smoking, alcohol, BMI. | 12y FU. | A linear association across sitting groups  1) almost  none of the time, 2) ~1/4, 3) ~1/2, 4)~ 3/4 or 5) almost all of the time.  Total sample HRs 1.01, 1.11, 1.36, 1.54 fully adjusted. |
| Overall |  |  |  |  |
| Dunstan et al. 2010 | HR 1.08 (95% CI, 1.00-1.17); fully adjusted.  Compared with 0-2 h/d of TV, HR 1.13 (0.87-1.36) for 2-4 h/d;  1.46 (1.04-2.05) for > 4 h/d; fully adjusted.  Examined the associations after excluding individuals who died within the first, second, third, and fourth years of follow-up. Strength and direction comparable to the original associations. | Australian men and women aged > 24 yrs (n=8,800).  Adjusted for age, sex, leisure-time exercise, waist circumference,  smoking, education,  total energy intake, alcohol intake, diet, BMI, hypertension,  total cholesterol, HDL-C, serum triglycerides, lipid lowering  medication use, previously reported CVD, glucose  tolerance status | 6.6 y FU. | Linear association with continuous variable  Categorical: 3 categories, increase in HR across categories, but not different from reference category. |
| Overall |  |  |  |  |
| Patel et al. 2010 | Women:  RR 1.13 (3-5 sitting/d); 1.34 (>6h/d) vs. 0-3h/d.  Men:  RR 1.07 (3-5 sitting/d); 1.17 (>6h/d) vs. 0-3h/d.  Sensitivity analysis among participants who are retired or homemakers; no differences. | US adults aged 50–74 yrs in 1992 (n=53,440 men and 69,776 women). Association stronger in women than in men.  Adjusted for age , race, marital status, education, smoking status, body mass index, alcohol use, total caloric intake, total PA, comorbidities score.  Stratified for BMI, similar findings, although association less strong as BMI goes up.  Excluded people who died in first year of follow-up. | 14y FU | Only 3 categories; significant difference between top two categories and reference category for both women and men, but stronger trend for women. |
| Overall |  |  |  |  |
| Wijndaele et al. 2010 | HR=1.05 per additional hour of TV/day  Excluded those with NCDs from analysis    Excluded those who died in the first 2 years | Regional UK men and women (n=13,197); adjusted total physical activity energy, EDU, smoking, alcohol, anti-hypertensive and lipid-lowering medication use, participant and family history of disease and total energy intake. | 9.5y FU. | Slope increases more after 3h/d. |
| Overall |  |  |  |  |
| Stamatakis et al. 2011 | Compared to <2 hrs/day:  HR 1.14 (0.80–1.62) for 2-4 hrs  HR 1.48 (1.04–2.13) >= 4 hrs.  p for trend 0.029  Sitting continuously: fully adjusted  HR 1.0011 (1.0005-  1.0017) per hr increase.  Excluded CVD events during the first year of follow-up and cancer registrations before baseline. | Scottish men and women aged >34 years (n=4,512).  Screen time adjusted for MVPA, sex, age, ethnicity, BMI, social class,  doctor-diagnosed diabetes and hypertension, longstanding  illness, marital status, smoking, occupational physical activity. | 4y FU. | Not linear. 2-4h/d screen time HR=1.14 (NS). Only effect for >4h/d. |
| Overall |  |  |  |  |
| Koster et al. 2012 | HR across quintiles of sedentary behaviour:  1 (lowest): 1.00  2: 1.74 (NS)  3: 2.74  4 (highest): 3.26.  Stronger results for percent time sedentary:  1 (lowest): 1.00  2: 1.53 (NS)  3: 4.05  4 (highest): 5.94. | US men and women from objectively assessed sedentary time in NHANES (n=1,906).  Sedentary behaviour adjusted for MVPA. | 2.8y FU. | Increase in effect from lowest to highest SB categories (see ‘strength’ column). |
| Overall |  |  |  |  |
| Matthews et al. 2012 | HRs for overall sitting, adjusted for MVPA:  <3h/d: 1.00  3-4: 0.98 (NS)  5-6: 1.03 (NS)  7-8: 1.02 (NS)  >9h/d: 1.19  HRs for TV viewing, adjusted for MVPA:  <1h/d: 1.00  1-2: 1.04 (NS)  3-4: 1.14  5-6: 1.31  >7h/d: 1.61 | US adults from 6 states and 2 cities; aged 50–71y (n=240,819).  Sedentary behaviour adjusted for MVPA and diet. | 8.5y FU. | Effect for TV, but only significant for highest level of overall sitting. |
| Overall |  |  |  |  |
| Pavey 2012 [full published citation now 2015] | Compared to <4h/day:  HR for 4-8 hrs/day 0.90 (0.79-1.03)  HR for 8-11 hrs/day 1.21 (1.01-1.44)  HR for >=11 hrs/day 1.24 (0.98-1.56)  Sensitivity analyses omitted deaths within the first 2 years. | Australian women aged 76–81 yrs (n=6,656).  Adjusted for age, education, marital status, area, smoking, alcohol consumption, BMI, physical activity, number of chronic conditions, self-reported health and assistance with daily tasks.  When stratified for PA, only significant association in those not meeting PA guidelines for those sitting >= 8 hrs. | Follow up 9 yrs (median 6 yrs) | 4 categories, HR for trend 1.03 (1.01 to 1.05) (see ‘strength’ column) |
| Overall |  |  |  |  |
| Van der Ploeg 2012 | HR:  0-4h/d sitting: 1.00 (ref)  4-8h/d: 1.02 (0.95-1.09) (NS)  8-11h/d: 1.15 (1.06-1.25)  >11h/d: 1.40 (1.08-1.15).  Analysis repeated including only subjects with more than 1y of follow-up. Results comparable. | Men and women from 45 & Up Study in NSW, Australia (n=222,497).  Sitting time adjusted for MVPA as well as sex,  age, educational level, marital status, urban or rural residence, BMI, smoking status, self-rated health, and receiving help with daily tasks for a  long-term illness or disability.  Interaction effects showed consistent results across sub-groups (e.g., sex, PA). | 2.8y FU. | Not linear. 4 categories. Effects stepped and significant from 8h/d (see ‘strength’ column). |
| Overall |  |  |  |  |
| Campbell 2013 | Pre-diagnosis sitting time. Compared to <3 hrs/day:  HR 1.13 (0.97-1.32) for 3-<6 hrs/day  1.36 (1.10-1.68) for >= 6 hrs/day.  Post-diagnosis sitting time. Compared to <3 hrs/day:  HR 1.13 (0.91-1.40) 3-<6 hrs/day  1.27 (0.99-1.64) >= 6 hrs/day.  Sensitivity analysis excluded deaths that occurred within 2 years of colorectal cancer diagnosis, deaths that occurred within 2 years of returning a survey, participants who were still employed, and participants whose post-diagnosis surveys were returned within 1 year of cancer diagnosis; no substantive differences. | Small US sample of older adults (n=2,293); cancer survivors.  Adjusted for age at diagnosis; sex; smoking status; body mass index; red meat intake and education. | Mean follow-up time from cancer diagnosis to death or end-of-study was 6.8 years. | Unclear Linear relationship (see ‘strength’ column).  Only 3 categories; only significant for one category for pre diagnosis. No differences for post diagnosis. |
| Overall: (Total sitting time) |  |  |  |  |
| Overall: (TV) |  |  |  |  |
| Chau 2013 | Total sitting time HR:  <4h/d: 1.00 (ref)  4-7h/d: 1.12 (0.89-1.42)  7-10h/d: 1.18 (0.90-1.57)  >10h/d: 1.65 (1.24-2.21).  TV time:  <1h/d: 1.00 (ref)  1-3h/d: 0.98 (0.75-1.27)  >4h/d: 1.11 (0.83-1.48).  Analyses excluded participants with less than 1 year of follow-up; results unchanged. | Central Norwegian men and women >19 years (n=50,817). Sedentary measures adjusted for meeting PA guidelines.  Adjusted for sex, BMI, education, smoking status, general health status, cardiometabolic disease status. | 3.3y FU. | Total sitting time: only highest level was significant.  TV: Not significant. |
| Overall |  |  |  |  |
| Kim 2013 | Total sitting time:  Men (HR):  <5h: 1.0  5-10h: 0.99  >10h: 1.04 (NS)  Women:  <5h: 1.0  5-10h: 0.99  >10h: 1.11 (p<.01)  TV time (>5 v <1h/d): HR=1.19 (men) & 1.32 (women) | US multi-ethnic cohort (n=134,596). Results differ by sex, and no effect for TV for Japanese American.  MVPA and other confounders included.  Those who reported a personal history of cancer, heart  attack or stroke at baseline and participants who died within the first year after cohort entry were excluded. | 13.7y FU. | Not for men. Women: some support; see ‘strength’ column. Effect for >10h total sitting only. |
| Overall |  |  |  |  |
| Leon-Munoz 2013 | Compared to sitting time >median in 2001 and 2003, newly sedentary: HR 0.91 (0.76-1.10);  formerly sedentary: 0.86 (0.70-1.05);  consistently sedentary: 0.75 (0.62-0.90).  Results similar after excluding those who died in the first 2 yrs of follow-up. | Spanish > 59 years (n=2,635).  Adjusted for sex, age, education, smoking, alcohol, BMI, physical activity, chronic lung disease.  Similar results were observed across strata defined by obesity,  morbidity, or functional limitations | 8 year FU. | 4 categories; only those consistently not sedentary show significantly lower ACM. |
| Overall |  |  |  |  |
| Martinez-Gomez 2013 | >8h/d sitting time v <8h/d: HR=0.70 (fully adjusted model). | Spanish adults aged >59yrs (n=3,465).  Analyses showed no changes when excluding those deceased within 2 yrs.  MVPA and other confounders included. | 9y FU | Only two levels of sitting tested. |
| Overall |  |  |  |  |
| Seguin 2014 | Compared to <= 4 hrs:  HR 1.03 (0.97-1.10) for >4-8 hrs  1.07 (0.99-1.14) for >8-11 hrs  1.12 (1.05-1.21) for >= 11 hrs.  Sensitivity analyses were conducted to examine a). effect of only sitting time (rather than combined, total sedentary time) and  b). effect of eliminating first 2 years of mortality data. Similar results. | US post-menopausal women aged between 50 and 79 years (n=92,234).  Adjusted for physical activity, age, race/ethnicity, education, marital status, BMI, self-rated health, smoking, alcohol, number of chronic diseases, and other confounders. | 12 y FU. | 4 categories; only one significant different from reference category, but trend was significant. |
| **Paper summary** (k=17; 2 ratings for Chau, k=18:  Green: 12 (67%)  Amber: 6 (33%) | Green: 13 (72%)  Amber: 5  Red: 0 | Green: 5  Amber: 13 (72%)  Red: 0 | Green: 12 (67%)  Amber: 5  Red: 1 | Green: 6  Amber: 10 (56%)  Red: 2 |

Abbreviations: ACM: all-cause mortality; BMI: body mass index; CI: confidence interval; HR: hazards ratio; LTPA: leisure-time physical activity; MVPA: moderate-to-vigorous physical activity; PA: physical activity; RR: relative risk ratio; SB: sedentary behaviour; FU: follow-up
